# The therapeutic mavericks: Potent immunomodulating chaperones capable of treating human diseases

**DOI:** 10.1111/jcmm.17669

**Published:** 2023-01-18

**Authors:** Paul Eggleton, Jorge De Alba, Michael Weinreich, Perry Calias, Roly Foulkes, Valerie M. Corrigall

**Affiliations:** ^1^ Revolo Biotherapeutics New Orleans Louisiana USA; ^2^ University of Exeter Medical School Exeter UK; ^3^ Centre for Inflammation Biology and Cancer Immunology, King's College London, New Hunts House Guy' Hospital London UK

**Keywords:** anti‐inflammatory, autoimmunity, cancer, myeloid cells, tolerance

## Abstract

Two major chaperones, calreticulin (CRT) and binding immunoglobulin protein (GRP78/BiP) dependent on their location, have immunoregulatory or anti‐inflammatory functions respectively. CRT induces pro‐inflammatory cytokines, dendritic cell (DC) maturation and activates cytotoxic T cells against tumours. By contrast, GRP78/BiP induces anti‐inflammatory cytokines, inhibits DC maturation and heightens T‐regulatory cell responses. These latter functions rebalance immune homeostasis in inflammatory diseases, such as rheumatoid arthritis. Both chaperones are therapeutically relevant agents acting primarily on monocytes/DCs. Endogenous exposure of CRT on cancer cell surfaces acts as an ‘eat‐me’ signal and facilitates improved elimination of stressed and dying tumour cells by DCs. Therefore, therapeutics that promote endogenous CRT translocation to the cell surface can improve the removal of cancer cells. However, infused recombinant CRT dampens this cancer cell eradication by binding directly to the DCs. Low levels of endogenous BiP appear as a surface biomarker of endoplasmic reticulum (ER) stress in some types of tumour cells, a reflection of cells undergoing proliferation, in which resulting hypoxia and nutrient deprivation perturb ER homeostasis triggering the unfolded protein response, leading to increased expression of GRP78/BiP and altered cellular location. Conversely, infusion of an analogue of GRP78/BiP (IRL201805) can lead to long‐term immune resetting and restoration of immune homeostasis. The therapeutic potential of both chaperones relies on them being relocated from their intracellular ER environment. Ongoing clinical trials are employing therapeutic interventions to either enhance endogenous cell surface CRT or infuse IRL201805, thereby triggering several disease‐relevant immune responses leading to a beneficial clinical outcome.

## 1 INTRODUCTION

**FIGURE 1:**
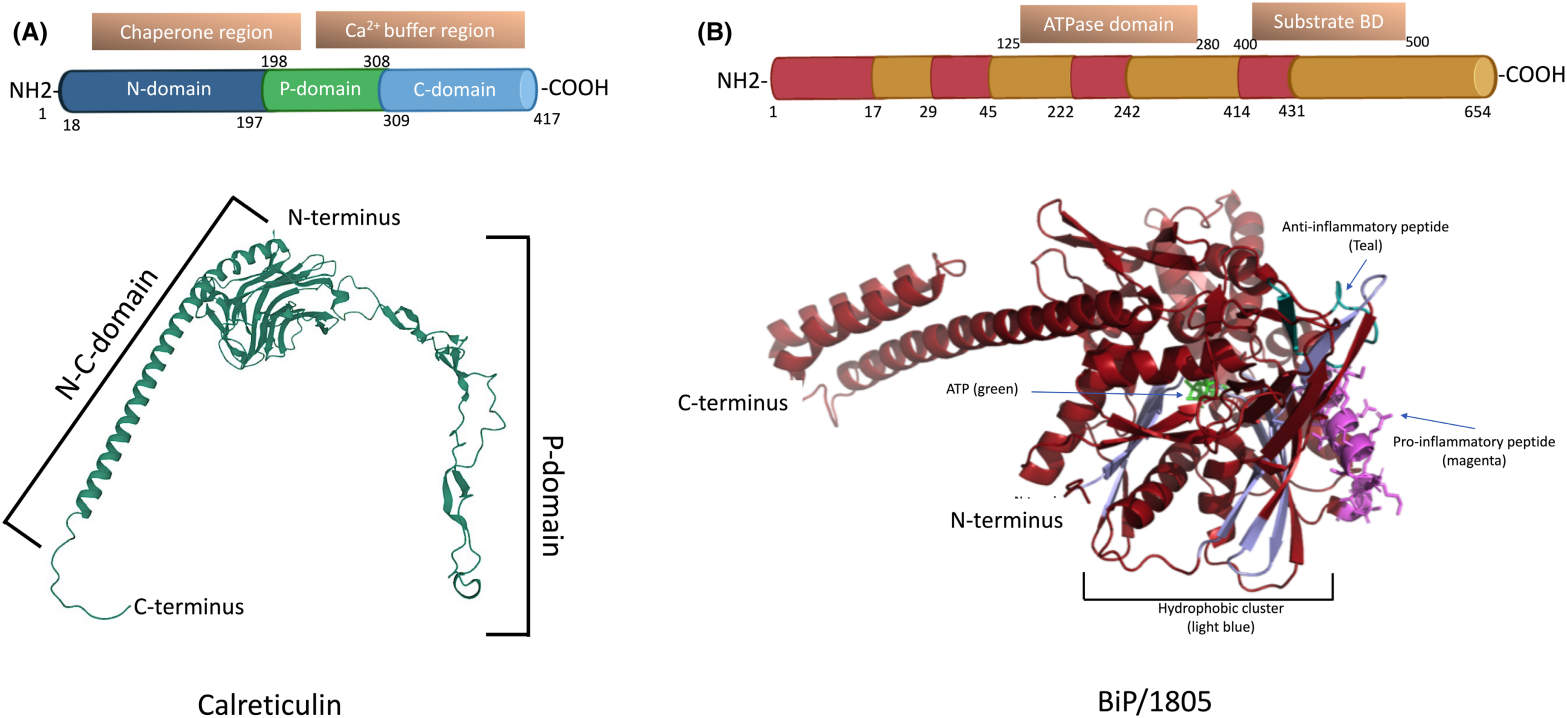
Schematic representations of calreticulin and GRP78/BiP (A) The amino acid sequence of CRT comprises of 417 amino acids, with leader sequence (aa 1–17), N‐domain (aa 18–197), a hair‐pin looped P‐domain (aa 198–308) and C‐domain (aa 309–417). The protein structure prediction of CRT presented is the AlphaFold2 model for CRT (AF‐P27797‐F1) (http://marrvel.org/human/gene/811). (B) The GRP78/BiP protein structure was prepared by PyMOL software V2.5.1 (PDB 5E84), the nucleotide‐binding domain is in the lower region of the protein model, while the substrate‐binding domain is situated towards the upper part of the molecule.

All organisms require a quality control system to assist in protein holding, folding, modification, degradation and for transportation of mature proteins to other organelles or secretion from the cell. The chaperones are a group of proteins that fulfil these roles and ultimately prevent nonspecific aggregation of unfolded proteins. Many molecular chaperones are also termed heat shock proteins (HSPs); they were first discovered in *Drosophila* and bacteria^1^ and become abundant under some environmentally stressful conditions, such as higher than normal temperature, pH or glucose fluctuation and hypoxic conditions. These conditions are prevalent in many diseases where there is infection, chronic inflammation, solid tumour growth or metabolic imbalance. In such harsh conditions, it is important that essential proteins are produced and correctly folded for the survival of the organism. In humans, most chaperones are believed to be located at some time in the ER and more infrequently in the cytosol, nucleus, lysosomes and mitochondria,^2^ where they have numerous and distinct functions. Chaperones have historically been allotted into HSP families based on their molecular weight, for example HSP10, HSP27, HSP40, HSP60, HSP70, HSP90, HSP110 and chaperonins.^2^ In the ER, GRP78/BiP (a HSP70 family member) is an ATP‐dependent chaperone, while calreticulin (gene name: CALR or protein name: CRT) is a Ca^2+^ dependent lectin chaperone (Figure 1). Common dogmas imply that chaperones are located and exclusively function solely within cells. But we and others have found that some chaperones including GRP78/BiP^3^, ^4^, ^5^, ^6^, ^7^ and CRT have immune‐modulating functions outside of the cell.^8^, ^9^, ^10^ Moreover, whereas CRT protein expression changes in response to raised cell temperature, GRP78/BiP is not particularly responsive to heat shock.

Eventual release of chaperones into the extracellular environment can occur either in a highly regulated manner or simply during cell necrosis. Individually, extracellular CRT^11^, ^12^, ^13^, ^14^, ^15^, ^16^ and GRP78/BiP^17^, ^18^, ^19^ have been monitored as biomarkers on the cell surface or in the serum/plasma, or other fluids of the body of patients with various diseases (see Table 1). Once outside the cell, CRT^11^ and GRP78/BiP^20^ have both been detected in healthy human plasma/serum at low nanogram/ml levels and often at higher concentrations in plasma/serum concentrations in various diseases. However, central to understanding of these chaperones is that their increased expression occurs because of disease pathology and not the cause. What is evident is that CRT and GRP78/BiP appear to provoke or restrain immune responses to diseases, once in the extracellular environment.^21^

**TABLE 1:** Cell surface/extracellular CRT or GRP78/BiP associated with human disease

Tumour type	Observations	References
Calreticulin—cell surface		
Colorectal cancer	CRT expression in colon cancer promoted infiltration of CD45RO^+^ memory T cells and enhanced 5‐year survival rates in patients	127
Nonsmall cell lung cell carcinoma	Loss of CRT mRNA expression negatively affects T‐cell immunosurveillance reducing patient survival	128
Acute myeloid leukaemia	Surface CRT on malignant blasts associated with increased CD4^+^/CD8^+^ Teffs and NK cells	129
Ovarian cancer	High CRT levels are associated with a TH1‐polarized, cytotoxic CD8^+^ T‐cell response	130
Calreticulin—plasma/serum		
Rheumatoid arthritis	Serum mean 4.87 ng/ml (*n* = 70) Mean controls 3.72 ng/ml (*n* = 35)	16
SLE	Serum mean 4.74 ng/ml (*n* = 80) Mean controls 3.70 ng/ml (*n* = 60)	53
MPN	Plasma median, MPN 5.2 ng/ml (*n* = 30) Plasma median controls 1.8 ng/ml (*n* = 10)	12
GRP78/BIP—Cell surface		
Tamoxifen‐resistant Breast cancer cell	The proline‐rich region—^638^AGPPPT^643^ of CD44v3 in SBD binds to GRP78/BiP in ER and is essential for translocation of GRP78/BiP to the cell surface MCF7‐LR cells	91
Human EndoC‐βH1 cells and primary islets	GRP78 is shuttled via the anterograde secretory pathway, through the Golgi complex and secretory granules, and identify the DNAJ homologue subfamily C member 3 (DNAJC3) as a GRP78‐interacting protein that facilitates its membrane translocation	131
GRP78/BIP—plasma/serum/saliva		
Healthy controls	Serum mean 18.4 ng/ml (*n* = 10) Age range 21–28; 7 females, 3 males	132
Healthy controls	Serum mean 14.4 ng/ml (*n* = 50) Age 53.9 ± 19.1; 21 females, 29 males	133
Healthy controls	Mean 42.64 ng/ml (*n* = 32)	134
Healthy controls	Mean 0.035 ng/ml (*n* = 20) Age 48.9 ± 11.4: 8 females, 12 males	135
Chronic hepatitis B HBV‐DNA 80 and 1.7 × 10^8^ IU/ml	Serum mean 9.2–15.0 ng/ml (*n* = 60) Age 42.1 ± 16.0 years; 28 females, 32 males	133
New diagnosed TB	Serum mean 40.88 ng/ml (*n* = 29)	134
Sepsis	Serum mean 70 ng/ml (*n* = 14)	136
Sepsis + infected	Serum mean 208 ng/ml (*n* = 52)	137
RA (immunoblot)	Saliva 5.5‐fold more versus HC (*n* = 20)	138
RA (immunoblot)	Synovial fluid positive for GRP78/BiP 13/18 (72%) RA subjects versus 5/13 (38%) of other joint diseases	116
72.8% type 2 diabetes, 52.5% obese and 78.6% metabolic syndrome	Plasma median 743 ng/ml (IQR—606 ng/ml) (*n* = 405) 206 females, 199 males Mean age 60 (range 50–67)	20
Multiple myeloma	Bone marrow aspirates, mean ~4 ng/ml (*n* – 44)	139
Type II diabetes	Mean 0.21 ng/ml	140
Chronic kidney disease (stage 3)	Mean 0.078 ng/ml (*n* = 22) Age 58.9 ± 19.0: 10 females, 12 males	135

*Note*: General conclusions: Cell surface CRT and GRP78/BiP are prevalent on some types of tumours. Cell surface CRT on pre‐apoptotic cells acts as an ‘eat‐me signal’, cell surface GRP78/BiP acts as a stress indicator. Release of extracellular CRT is marginally raised in some cancer and autoimmune pathologies that may impact the ‘eat‐me’ capacity of DCs to clear apoptotic cells. By contrast, there are higher ng/ml extracellular GRP78/BiP levels reported in healthy control subjects that become elevated in numerous pathologies.

Abbreviations: DC, dendritic cell; MPN, myeloproliferative neoplasm; SBD: substrate‐binding domain; SLE, systemic lupus erythematosus.

### 1.1 Chaperone dogma and diversity of function

Chaperones function to aid protein folding and homeostasis in their intracellular environment. Once certain chaperones are released from cells, they take on ‘moonlighting’ functions, different from their normal intracellular role. For example, both extracellular CRT and GRP78/BiP appear to trigger several innate and adaptive immune responses. The effects are diverse and include the up‐, or downregulation of metabolic and immune mediators, that alter development or maturation of immune cells, such as monocytes and dendritic cells (DCs). However, there is mounting experimental data to suggest extracellular chaperones, for example CRT can behave as pattern recognition molecule that can promote pro‐inflammatory immune changes, whereas GRP78/BiP does not fit this paradigm and has been defined as ‘regulatory‐associated molecular pattern (RAMP)’ that in the extracellular environment can act to promote immediate anti‐inflammatory and pro‐resolution signals, and thus both chaperones can be exploited for therapeutic use.

### 1.2 Chaperone versatility

#### 1.2.1 Chaperone history and evolution

Interestingly, chaperone function spawned from a dogma that proteins folded spontaneously requiring little help from other proteins. In 1962, Anfinsen describing his ‘thermodynamic hypothesis’ concluded that the native form of a protein is its most thermodynamically stable configuration and side‐chain functional groups influenced the correct folding of proteins by the formation of intramolecular disulphide linkages.^22^ Later, it became apparent that a group of highly conserved chaperones were shown to be involved in the assembly and disassembly of intracellular proteins and were located in the ER, nucleus and cytoplasm of cells.^23^

From primitive single‐cell organisms to sophisticated human immune cells dealing with inflammation, chaperones are essential for protein folding. In humans, there are ~194 chaperones expressed and in whole blood 122 chaperones have been detected at ≥10 transcripts per million. Some core chaperones are ubiquitous, while others are associated with tissue‐specific functional networks. Many chaperones interact with other chaperones, which allows them to alter their function^24^ and as neurologists are acutely aware, when some chaperones in brain cells decline with age, cellular protein aggregates increase.^25^ Chaperones have evolved to be more versatile as they evolve from unicellular to multicellular organisms. The *Escherichia coli* chaperones are all constitutive but can be induced further upon environmental stress conditions. By contrast, human cells retain constitutive inducible chaperones; core chaperones conserved in all tissues (‘essential house‐keeping functions, e.g. UPR’), but others that can be differentially expressed across tissues (e.g. brain, lung, or muscle), giving chaperone a greater dynamic functionality in certain organs and cell types.^24^ CRT is a very ancient protein present in all flora and fauna except, yeasts or bacteria, suggesting it has evolved its biological functions over 350 million years.^26^ GRP78/BiP may be 400 million years old, as a homologue of this gene is present in yeast as KAR2 (*Saccharomyces cerevisiae*) and DnaK in bacteria, where its suppression leads to the inhibition of translocation of secretory proteins.^27^ Currently, ER chaperones (e.g. CRT, calnexin, protein disulphide isomerase [PDI] and GRP78/BiP) are known to have pivotal roles in protein folding, cell survival and development. Often, these functions require that chaperones be retained within the ER. To facilitate, retention, the C‐terminal KDEL amino acid sequence on chaperones, ensures that they are retained or recycled back into the ER.^28^ It is becoming apparent that the location of various chaperones influences their function. For example, intracellular CRT is essential for glycoprotein folding,^29^ but when transported to the surface of tumour cells can act as an ‘eat‐me’ signal on pre‐apoptotic cells,^30^ or inhibit complement activation.^31^ Once in the extracellular environment, it influences cellular apoptosis of immune cells^32^ and phagocytosis^33^, ^34^ or binds to stimulatory co‐factors such as LPS, which can influence immune regulation and inflammatory pathways as well as innate immunity, in multiple species.^35^, ^36^, ^37^ Similarly, GRP78/BiP has an essential role to play in the quality assurance of protein assembly and transport from the ER but can also translocate to the cell membrane of stressed cells where it can engage in anti‐ and pro‐survival functions. However, in an extracellular environment, it selectively binds and is rapidly internalized by myeloid cells—monocytes, macrophages and DCs where it can trigger a series of signalling pathways that dampen inflammation and restore immune homeostasis.^3^, ^4^, ^5^, ^6^, ^7^, ^38^

### 1.3 How chaperones are naturally released from cells

**FIGURE 2:**
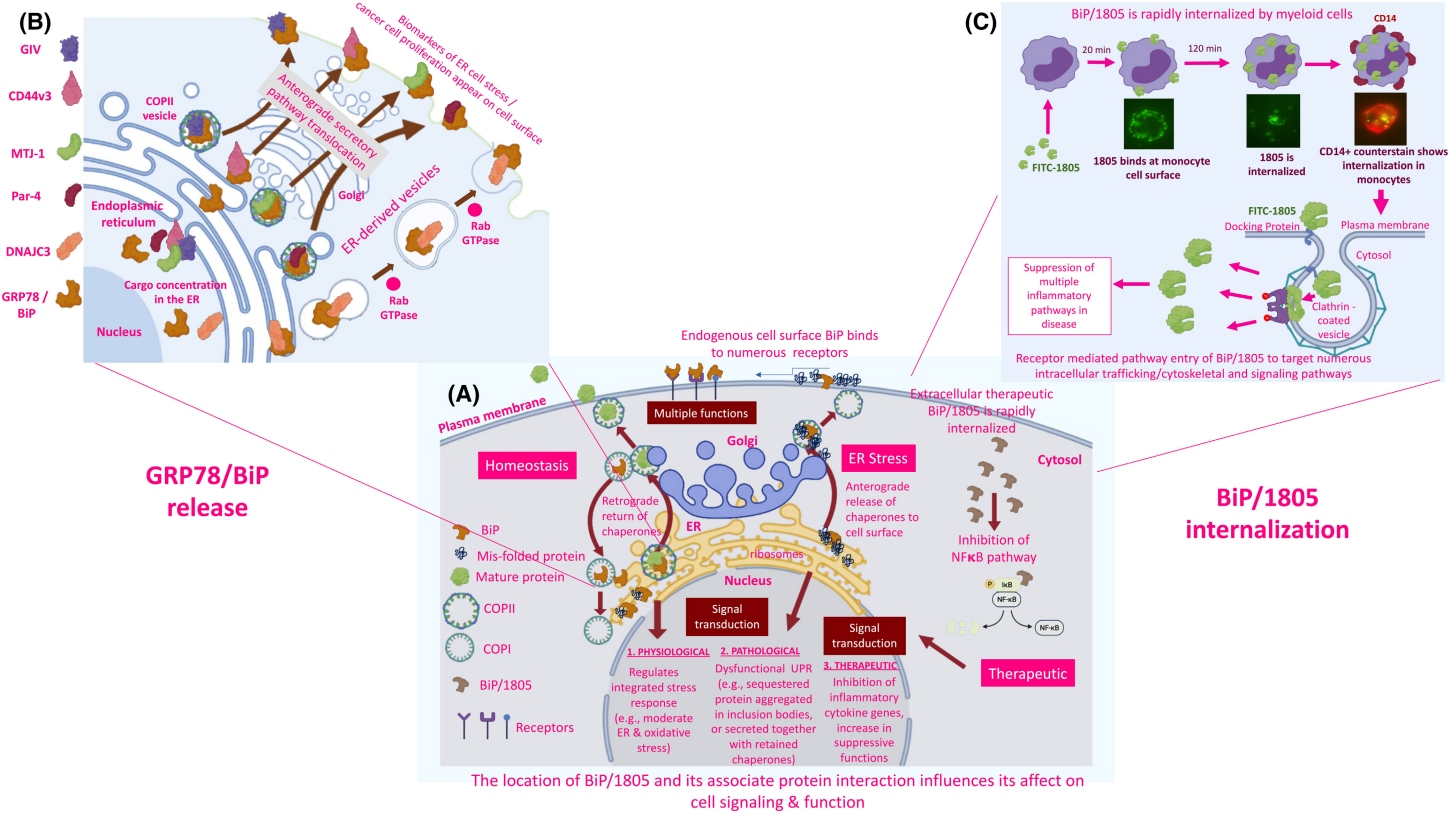
Exit of GRP78/BiP and entry of IRL201805 influences different cell signalling and functional pathways. (A) Under physiological conditions, the pH and Ca^2+^‐regulated endoplasmic reticulum (ER) serves as a location for nascent peptides to interact with various KDEL‐retaining chaperones where they undergo folding and insertion into COPII vesicles for transportation to the Golgi and secreted or inserted within the plasma membrane. During ER/oxidative stress, GRP78/BiP and KDEL‐containing chaperones (e.g. BiP or CRT) can aid protein transport to the Golgi as part of the integrated stress response (ISR) and are retained by the KDEL receptors in the acidic conditions and then returned to the ER in COPI vesicles by retrograde translocation. (B) Under severe stress, the KDEL‐containing chaperones bind to accumulated protein aggregates and travel with them from the ER to the Golgi. Here, the KDEL receptors become saturated and via this anterograde process, chaperones including BiP or CRT and aggregated proteins, are either retained in cytosolic inclusion bodies, or translocated to the cell surface where they bind to cell surface proteins/receptors or are secreted. (C) By contrast, the therapeutic exposure of cells to extracellular IRL201805 does not signal through the ER‐Golgi KDEL‐receptor pathways. It is rapidly internalized <2 h possibly by receptor‐mediated endocytosis. Once in APCs, IRL201805 has direct tolerogenic effects on the cells, such as increase IDO, decrease pro‐inflammatory cytokines, while increasing anti‐inflammatory cytokines. There is also evidence that endogenous GRP78/BiP self‐peptides are loaded in HLADR‐II molecules and presented on the cell surface, where they may activate tolerogenic Tregs against self‐peptides (see Section 3.5).

Proteins destined for secretion are synthesized by ribosomes and translocate to the ER. The newly synthesized proteins interact with ER chaperones; GRP78/BiP, calnexin, CRT and PDI. These chaperones aid the assembly of polypeptides into mature proteins and assist their transport initially to the ER‐Golgi intermediate compartment (IC). This processing and transportation of proteins are aided by chaperones such as CRT and GRP78/BiP, before the chaperones are returned to the ER via COPI vesicles, while the mature protein continues to the cell surface for insertion into the plasma membrane or secretion via the COPII vesicles (see Figure 2A). Although CRT and GRP78/BiP are chaperones with C‐terminal KDEL sequences, their mode of release from cells differs (see below). Upon certain types of cellular stress, some chaperones actively get released from the cells along with some of their binding partners (see below). Once released from cells, we know much less about how specific chaperones function outside of cells. We are interested in how ER chaperones influence the innate and adaptive immune system once in the extracellular environment.

## 2 EXTRACELLULAR CALRETICULIN

### 2.1 Release of CRT from human immune cells—First observations

Interest in the release of endogenous CRT has gained momentum given its recent identified roles in immunogenic cell death of tumours^39^ and myeloproliferative neoplasms (MNPs)^40^ Numerous studies have investigated how CRT is released from cells during cell stress, pharmacological intervention or due to somatic mutations.^41^ In 1994, Eggleton and colleagues observed CRT on the surface of neutrophils; stimulation with the bacterial tri‐peptide FMLP led to the release of CRT into the extracellular medium where it is was shown to bind to innate immune molecules C1q and collectins.^42^ Concurrently, a molecule on the surface of the monocytic cell line U937 which also bound to C1q and was named a ‘C1q receptor’ having the same sequence as CRT was identified.^43^ These unexpected observations of an apparent ER chaperone interacting with a complement protein were intriguing. Further work showed that CRT could bind to C1q and block ‘classical’ complement activation.^31^ Meanwhile, Pritchard and co‐workers also observed CRT‐like molecule on the surface of a persistent hookworm parasite—*Necator americanus* collected from Papua New Guinea.^44^ They hypothesized and later confirmed that the presence of CRT on the surface of the parasite evolved as a mechanism by which hookworms avoid attack by the host's complement system.^45^ More recently, work by Arturo Ferreira and his team have demonstrated that other parasites, particularly *Tryanosoma cruzi*, also present CRT on their cell surface to evade attack by the complement system.^46^ This early work provoked an interest to study CRT—immune interactions further but did not explain how CRT cell surface expression was regulated either in humans or more primitive species.

### 2.2 Extracellular CRT release is associated with autoimmunity

A link between immune cells, surface CRT and complement attracted interest when it became evident that dead and dying cells in the circulation were being cleared in a noninflammatory manner by a process of apoptosis and that changes in CRT expression were associated with this process.^47^ It was shown that complement proteins were important in clearing apoptotic cells from the circulation, because a rare deficiency in which individuals lacked C1q led to the development of a severe autoimmune disease, systemic lupus erythematosus (SLE). Meanwhile, Walport and colleagues showed that C1q deficiency led to defective apoptosis in these patients.^48^ It was proposed that extracellular CRT acted as an intermediary, binding to C1q on the surface of apoptotic cells, while also docking with CD91 on professional phagocytes, thereby promoting uptake of cell debris by micropinocytosis.^49^ Some former clues as to how CRT is released from cells came from the observations of Sontheimer, who observed that CRT transcriptional activity increased when dermal epithelial cells were stressed by a calcium ionophore, heat shock or heavy metals such as zinc and cadmium.^50^ Sontheimer proposed that CRT was a HSP and, like GRP78/BiP, was overexpressed under certain cell stress conditions. Similarly to GRP/BiP, extracellular CRT could influence a number of immune pathways, for example inhibit complement activation and,^31^ alter DC recognition and uptake of tumour cell antigens.^51^ More recently, mutant forms of extracellular CRT have been shown to bind to a thrombopoietin (TPO) receptor, precipitating the induction of rare blood cancers called MPNs, in which stem cells in the bone marrow make excessive numbers of various blood cells.^52^ In SLE, anti‐CRT antibodies are detected and serum CRT protein levels are associated with disease activity, particularly nephritis damage^53^ and RA.^16^ In addition, citrullinated CRT, which is overabundant in the RA synovium, potentiates HLA‐DRB1 share epitope signalling leading to increase bone erosion, PAD activation and raised TNF‐alpha serum levels in experimental models of RA.^54^, ^55^

### 2.3 Somatic mutant CRT is associated with myeloproliferative disorders

CRT, like GRP78/BiP, normally has a negatively charged C‐terminal KDEL sequence (see Section 3.2), which aids CRT docking into the positively charged binding cavity of the KDEL receptor.^56^ These biophysical features of CRT are an important aspect of the KDELR chaperone retention system to retain chaperones internally. This has been underscored in the last decade or so with human MPNs, where the frameshift in mutant CRT's produced, results in mutant proteins with positively charged C‐termini. The MPNs are a rare group of blood cancers originating in the bone marrow in which the body makes too many of a particular type of blood cells. Somatic changes in CRT were originally observed in patients with essential thrombocythemia (ET) and primary myelofibrosis (PMF), which are both MPNs.^41^, ^57^ In these neoplasms, ~30–50 mutations have been found in CRT. Two common mutations consist of a 52 amino acid deletion (type I mutation) or a five amino acid insertion (type II mutation) in the C‐terminus. In all cases, the ER retrieval signal (KDEL) is lost in the C‐terminus region of the protein allowing these mutants to escape ER retention.^40^ In a study of MPN patients (*n* = 113) carrying the mutant CRT, the KDEL‐lacking mutant CRT was secreted into their plasma, with a mean concentration of ~24.6 ng/ml (range 0–156.5 ng/ml).^58^ The mutations can lead to structural changes of the protein within the ER. Two positively charged C‐domains have been shown to form a homodimer and a hypothetical model proposed, in which the homodimers may intertwine to form a dimeric complex, which facilitates the N‐domains to bind to N‐glycans on the thrombopoietin (TPO/MPL) receptor.^58^, ^59^ Normally, the P‐domain of the protein can prevent this interaction.^60^ The mutant CRT–MPL complex moves to the cell surface via the secretory pathway. Once embedded in the plasma membrane, the mutant CRT lodges within the thrombopoietin receptor causing constitutive activation of Janus kinase 2 (JAK2)/STAT5 signalling pathway, which is pathogenic and promotes oncogenesis. Recent work has revealed cells presenting with both the TPO receptor and mutant CRT complex are hypersensitive to additional exposure to exogenously released mutant CRT. Indeed, the exogenous mutant CRT found at levels observed in MPN patients, acts as a ‘rogue’ cytokine, capable of activating TPO receptor/JAK–STAT signalling in patient primary cells, by binding to the immature N‐glycosylated TPO receptor complexed with mutant endogenous mutant CRT.^61^

### 2.4 Cell surface CRT and Immunogenic cell death (ICD)

The work of the Kroemer group, studying murine and human tumour cells, identified several ways to explain how CRT translocates onto the cell surface and emphasized the importance of the timing of CRT release. Cancer cells successfully form and sustain tumours because they are host cells and avoid scrutiny by central thymic tolerance by keeping certain damage‐associated molecular patterns (DAMPs) to a minimum on their cell surface. However, it has been proposed that some DAMPs promote tumour progression, while others can promote immune‐mediated inhibition of cancer and this can add to the complexity of DAMPs in tumorgenesis.^62^ DAMPs associated with cancers can arise from numerous cell sites, including the nucleus (e.g. high‐mobility group box 1 protein: HMGN1), mitochondria (e.g. ATP and DNA), cytoplasm (e.g. F‐actin) and endoplasmic reticulum (ER; e.g. CRT).

Kroemer and his team focused on the translocation of CRT to the cell surface on tumour cells during ER cell stress and apoptosis. But, it became clear that the translocation of CRT to the cell surface is not straight forward and it appears that several different stimuli‐dependent signalling pathways can be elicited in which CRT can reach the cell surface under pre‐apoptotic conditions^63^, ^64^, ^65^, ^66^; thus, it was proposed for surface CRT to provoke an immune response on tumour cells; it had to be released on pre‐apoptotic cells. Not surprisingly, human cells in a necrotic state or in the early stages of apoptosis due to ER‐Ca^2+^‐dysregulation also present CRT on their cell surface or release CRT into the extracellular milieu.^33^, ^67^ To this end, the term immunogenic cell death (ICD) is used to distinguish the immune recognition function of surface CRT on pre‐apoptotic cells versus CRT was found on the surface or released from necrotic or apoptotic cells. It is fortuitous that chemotherapeutic drugs such as anthracycline antibiotics and platinum‐based platins act on many metabolic pathways in cells, including ER stress. Like many chemotherapeutic drugs, they are relatively toxic as such, they trigger ER cell stress particularly in rapidly proliferating tumour cells. This leads to ER stress proteins being activated, including CRT. A series of signalling events occur, resulting in phosphorylation of the eukaryotic translation initiation factor eIF2‐α by the PKR‐like ER kinase (PERK), followed by proteolytic cleavage of ER‐sessile protein BAP31 by caspase‐8, and activation of proapoptotic proteins BAX and BAK. This leads to an anterograde release of CRT from the ER to the Golgi apparatus and exocytosis of CRT‐containing vesicles to the plasma membrane.^66^ Vesicle‐bound SNARE proteins that facilitate vesicle fusion aid this translocation. The notion that CRT translocates to the plasma membrane on its own is unlikely, as it is often associated with other proteins, for example ERp57.^64^, ^68^ Interestingly, other more physical cancer cell therapies, such as photodynamic treatments (PDT), can similarly lead to CRT appearing on the cell surface but do so independently of eIF2‐α phosphorylation or association of ERp57. This suggests there are numerous heterogenous vesicular transport pathways that may aid CRT presentation on the surface of pre‐apoptotic cells.

Cell surface‐bound CRT as opposed to extracellular CRT is considered the dominant ICD signalling molecule on tumour cells. Cell surface CRT is believed to disturb the balance between the CD47 (‘don't eat me’) signals with SIRPα on phagocytes, especially DCs. The exon‐9 mutated CRT can be released via the anterograde ‘Golgi‐secretory pathway’.^69^ Alternatively, CRT can be released from necrotic cells in various pathological conditions (Table 1) or after chemotherapy. In addition, professional phagocytes such as monocyte/macrophages, that scrutinize and eliminate tumour cells, are known to release CRT via activation of Bruton's tyrosine kinase (BKT/TLRs) pathway.^70^ CRT phosphorylation by BKT in macrophages is important for CRT trafficking to the cell surface to function as a bridging molecule as part of the CRT/CD91/C1q complex, which initiates phagocytosis of apoptotic cells.^71^ Some patients with tumours are nonresponders to chemotherapeutic treatments that normally induce transport of CRT onto their tumour cell surface. Lin et al.^72^ have studied cancer patients who do not respond to checkpoint inhibitors and revealed that they possess high expression of the gene coding for stanniocalcin‐1 (STC1) that is associated with poor survival. They demonstrated that intracellular STC1 binds cytosolic CRT keeping it near to mitochondria and preventing CRT translocation to the cell surface.

## 3 EXTRACELLULAR GRP78/BIP/1805

Endoplasmic reticulum chaperones such as CRT and BiP are both capable of inducing adaptive immune responses once they are released from cells. There are, however, notable differences in how these two chaperones function. CRT promotes maturation of APCs, cross‐presentation of tumour antigens and facilitates recruitment of Teffs, primarily CD8 cytotoxic T cells, that provide a robust immune response to tumours (Figure 4B). For CRT, to drive this anticancer immunity, it requires release and membrane binding of endogenous CRT from pre‐apoptotic cancer cells. By contrast, pharmacologically manufactured homologues of BiP, for example IRL201805 (Table S1) but, not release of endogenous BiP, promotes induction of tolerogenic APCs and promotes suppressive features of T‐regulatory cells (Figure 4A). The pharmacokinetics (PK) and pharmacodynamics (PD) also differ. Cell surface CRT is retained on the surface of tumour cells for days, until recognized by antitumour cells, and then, the cells are eliminated, representing a long PK, short PD. When IRL201805 is administered in vivo, it has a short serum half‐life (1–4 h), but long PD > 12 weeks. Consequently, while cancer immunologists have focused on ways to manipulate release of endogenous CRT from cells, immunologists interested in preventing autoimmunity and resolving inflammation have focused on delivering extracellular IRL201805 to immune cells, for internalization and activation of immunosuppressive features of myeloid and T cells. For both these proteins, a comprehensive knowledge of how they respond with cells under resting and stress conditions is important, as this will aid our understanding of how long‐term immune responses are triggered during stress conditions (e.g. tumour production or autoimmunity) before re‐establishing a homeostatic rebalance upon alleviation of pathological conditions.

### 3.1 Extracellular GRP78/BiP interaction with immune cells—First observations

In the 1990s, the discovery of elusive initiating autoantigens in RA was a focus for research. In a proteomics approach, denaturing polyacrylamide gel electrophoresis and immunoblotted were used to separate soluble proteins from human chondrocyte lysates. These blots were screened with RA or healthy/disease control sera and the antigen–antibody bands visualized with enhanced chemiluminescence. Those of interest were then subject mass spectrometry, which identified a 72 kD protein, GRP78/BiP.^38^ After preparation of a recombinant human BiP (RhuBiP), we investigated whether there was any possibility that the antigen was arthritogenic. Even when co‐administered with Freund's complete adjuvant, there was no sign of increased joint inflammation in different animal species. However, when administered intravenously either before induction of murine collagen‐induced arthritis (CIA) or, at the point of disease onset, animals were protected from development and progression of disease. Latterly, it was discovered BiP/1805 was protective in adjuvant arthritis in rats^38^ and a human TNF transgenic mouse model.^5^ In RA, GRP78/BiP is found in serum and synovial fluid, and it is possible that the high concentration of GRP78/BiP in synovial fluid breaks tolerance. Synovial fluid T cells show a recall antigen response to GRP78/BiP.^38^ Confirmation of this immune activation comes from the presence of autoantibodies to GRP78/BiP in the sera and synovial fluid, indicating the cells have been activated by self‐antigen.^73^

The release of GRP78/BiP from cells in a number of diseases (Table 1) may be taken up by APCs, presented to T cells, allowing autoreactive T‐regulatory cells (Tregs) and T‐effector cells (Teffs) to be exposed to this self‐antigen.^74^ This might explain the antibody generation against GRP78/BiP observed too (Table 2).^38^ However, in chronic diseases, low‐dose exposure of self‐antigens such as GRP78/BiP is a mechanism by which peripheral tolerance is established by downregulating the pro‐inflammatory cells followed by autoreactive Treg production.^3^ Therefore, identifying diseases where endogenous GRP78/BiP is secreted above a certain threshold might provide an opportunity where a single or series of high therapeutic doses of IRL201805 could accelerate a sustained immune tolerance response. Interestingly, several diseases have been recorded in which extracellular GRP78/BiP is present in plasma at higher levels than age–sex match control subjects (Table 1).

**TABLE 2:** CRT and GRP78/BiP elicit autoantibody production^a^

Calreticulin		
Autoimmune disease	Antibody isotype	References
Refractory coeliac disease	IgA	141
Primary biliary cirrhosis	IgA	142
Inflammatory bowel disease	IgG	143

Cancers	Antibody isotype	References
Pancreatic cancer	IgG	144
Rheumatoid Arthritis/Bronchiectasis	IgG against citrullinated CRT	145

GRP78/BIP		
Autoimmune disease	Antibody isotype	References
Rheumatoid arthritis	IgG against 68 kDa fragment of 107/167—64%) subjects	146
Rheumatoid arthritis	IgG (252/400—63%^+ve^)	147
Rheumatoid arthritis/SLE	IgG	148
Rheumatoid arthritis	IgG	38
Rheumatoid arthritis	IgG against only carbamylated GRP78	149
Rheumatoid arthritis	IgG against GRP78/BiP_295–314_ R305citrulline and GRP78/BiP_500–519_ R510citrulline	150
SLE	IgG	151
Multiple sclerosis	IgG	152
COPD/atherosclerosis	IgG GRP78/BiP‐aa_246–260_	153
Type 1 diabetes	IgG against citrullinated GRP78/BiP^503^ DVNGILR^510^VTAE^514^	154
Type 1 diabetes	IgG (20/61—33%^+ve^)	108

Cancers	Antibody isotype	References
Colorectal carcinoma	IgG	155
Hepatocellular carcinoma	IgG	156
Gastric cancer	IgG	157
Prostate cancer	IgG against LIGRTWNDPSVQQDIKFL (Leu^98^‐Leu^115^)	93

Abbreviations: COPD, chronic obstructive pulmonry disease; SLE, systemic lupus erythematosus.

^a^ Anti‐CRT and anti‐GRP78/BiP are also found in healthy controls but at lower titres and in less frequency in most of the studies cited in this table. Studies of control subjects cells induced by stress also generate GRP78/BiP autoantibodies.^158^

### 3.2 GRP78/BiP release from cells via stress

GRP78/BiP, like several ER chaperone proteins, has a KDEL sequence that can engage with KDEL receptors. KDEL receptor 1 (KDELR) was originally found to be responsible for the return of soluble ER‐resident proteins to the ER from the IC of the cis‐Golgi. This retrograde transport requires soluble ER‐resident proteins to either have a KDEL‐like motif at their C‐terminus or to form a complex with ER‐resident proteins that do.^75^ In the epithelial HeLa cell line, it has been reported that KDELR modulates ER stress responses.^76^ More recent studies have suggested that KDELR function goes beyond motif recognition by demonstrating that the chaperone‐bound KDELR triggers the activation of Src family kinases at the Golgi complex, a phenomenon that may be critical for intracellular signalling cascades.^77^, ^78^ Integrated stress responses (ISR) are stress‐response programmes that coordinate activation of four stress kinases (double‐stranded RNA‐dependent protein kinase R [PKR], RNA‐dependent protein kinase‐like ER kinase [PERK] and eukaryotic initiation factor 2 [eIF2a] kinase general control nonrepressed 2 [GCN2]) to adjust cellular homeostasis by responding to various types of stress signals, including ER stress, amino acid deprivation, infection with double‐stranded RNA viruses, haem‐deficiency and oxidative stress (see Ref. [79]). As shown in Figure 2A, under physiological homeostatic conditions, the ribosome‐enriched ER is at neutral pH and in a calcium‐regulated environment, in which nascent peptides interact with various KDEL‐containing chaperones to enable correct folding and insertion into coat protein complex II (COPII) vesicles and transported to the Golgi for secretion or plasma membrane insertion. During moderate ER stress, GRP78/BiP and other soluble KDEL‐containing chaperones can accompany the proteins to the Golgi as part of the integrated stress response (ISR) and are retained by the KDEL receptors in the acidic conditions, which favours KDEL and receptor interactions, before returning to the ER in coat protein complex I (COPI) vesicles by retrograde translocation. Under pathological conditions during dysfunctional proteostasis, the KDEL‐containing chaperones bound to excessive protein aggregates, possibly binding to the KDEL region of the chaperone, are no longer retained in the ER and travel to the Golgi. Here, the KDEL receptors either become saturated or are unable to detect the KDEL‐containing chaperone–protein complexes and via an anterograde process, chaperones including GRP78/BiP and aggregated proteins are either retained in cytosolic inclusion bodies or translocates to the cell surface in COP II vesicles where they bind to cell surface proteins/receptors or are possibly secreted (Figure 2B). Alternatively, it is possible that the entry of extracellular soluble GRP78/BiP or, pharmacologically prepared IRL201805, into cells occurs by an entirely different endocytic pathway (Figure 2C) but not exclusively targeting myeloid cells and appears to target entry into and some specific subsets of immune cells thereby promoting a completely different set of functional consequences,^80^ which are detailed below (see Section 3.4).

### 3.3 GRP78/BiP is a biomarker of cell stress presented on the surface of some cancer cells

Resting cells do not generally present with cell surface GRP78/BiP, unless stressed. However, cells such as synovial cells from RA patients, tumour cells and most virally transformed cells can show surface expression and secrete GRP78/BiP into the local environs. Rapidly dividing cells, as occur in infection, inflammation or cancer, quickly exhaust the glucose, oxygen and other metabolites required for growth. This stress causes an imbalance of ER homeostasis resulting in the unfolded protein response as discussed above. Therefore, as a consequence of abnormal cell growth, GRP78/BiP can ultimately present on the cell surface of numerous cell types (Table 1) including, lymphoma, neuroblastoma, lymphoblastic leukaemia, ovarian tumour, lung and colon adenocarcinoma.^81^, ^82^, ^83^, ^84^, ^85^ One notable feature of GRP78/BiP that is required for its translocation to the cell surface is the substrate‐binding domain (SBD). This suggests GRP78/BiP translocates to the cell surface accompanied by partner proteins particularly on cancer cells. Prostate apoptosis response‐4 (PAR‐4) is a tumour suppressor molecule that can cause apoptosis in some cancer cells but not normal or transformed immortalized cells.^86^ GRP78/BiP and Par‐4 translocate to the cell surface together but how they do so is not clear. Par‐4 and GRP78/BiP may co‐localize within the ER prior to translocating to the plasma membrane.^87^ It is believed GRP78/BiP binds to the SAC (selective for apoptosis in cancer cells) domain of Par‐4.^88^ When Par‐4 is silenced by siRNA, less GRP78/BiP is seen on the surface of tumour cells.^89^ Other cancer cell types may rely on different GRP78/BiP binding partners to relay it to the cell surface, suggesting there is a degree of cell selectivity that influence GRP78/BiP translocation that needs further work. Other proteins have shown to be important in aiding translocation of endogenous GRP78/BiP to the surface of some tumour cells, such as MTJ‐1, which binds to GRP78/BiP in the ER and assists GRP78/BiP function as a chaperone. Silencing of the MTJ‐1 gene greatly reduces MTJ‐1 mRNA and protein levels subsequently abolishing cell surface localization of GRP78/BiP.^90^ CD44v as a transmembrane protein, involved in cell–cell interaction, adhesion and migration is synthesized in the ER and is translocated to the cell surface associated with GRP78/BiP under specific conditions.^91^ Finally, G alpha‐interacting vesicle‐associated protein (GIV) interacts with GRP78/BiP through its carboxyl‐terminal substrate‐binding domain (aa 341–654) during ER stress to promote its cell surface translocation. GIV‐depleted (GIV‐shRNA) HeLa cells demonstrated impaired GRP78 on the cell surface, indicating GIV plays a role in GRP78 transport to the cell surface.^92^ Once on the cell surface, GRP78/BiP has been reported to bind to numerous self‐antigens and microbial proteins and consequently has been proposed as a nonspecific ‘receptor’ for a multitude of proteins.^93^ Collectively, these independent studies reveal that GRP78/BiP can shuttle out of the cell with binding partners and relocate to the cell surface, where it becomes embedded in the plasma membrane.

### 3.4 Entry of extracellular GRP78/BiP/1805 and other HSPs into APCs by receptor‐mediated endocytosis

Evidence of HSP entry into murine monocytic (P388D1) and DC lines (D2SC/1) via receptor‐mediated endocytosis (RME) was demonstrated in the late 1990s. Arnold‐Schild et al. showed that immune‐modulating HSPs gp96 and heat shock cognate 70 (HSC70) were taken up myeloid APCs via clatherin‐coated pits that cluster receptors, bending the plasma membrane in discrete regions to become endosomal structures.^94^ Interestingly, in the same study, unlabelled HSPs in excess were unable to compete for endocytosis of a specific labelled HSP, suggesting each protein has its own specific receptor, although these were not identified. Proteins entering cells by RME use specific receptors and/or docking proteins to regulate specificity of entry into cells. A number of surface receptors have been identified that internalize HSPs, including scavenger receptors/CD91 (HSP70/90/110), toll‐like receptors (HSP27/60/70), which allow internalization of the HSPs, prior to peptide processing and presentation as antigen in surface MHC molecules to T cells, via their T‐cell receptors (TCR).^95^ However, there is no evidence that extracellular GRP78/BiP or IRL201805 binds to the above receptors, suggestive that IRL201805 may enter cells and engage with novel receptor(s), which is currently being investigated.

### 3.5 Internalization of GRP78/BiP immunomodulates both APC/B/T‐cell immune responses

**FIGURE 3:**
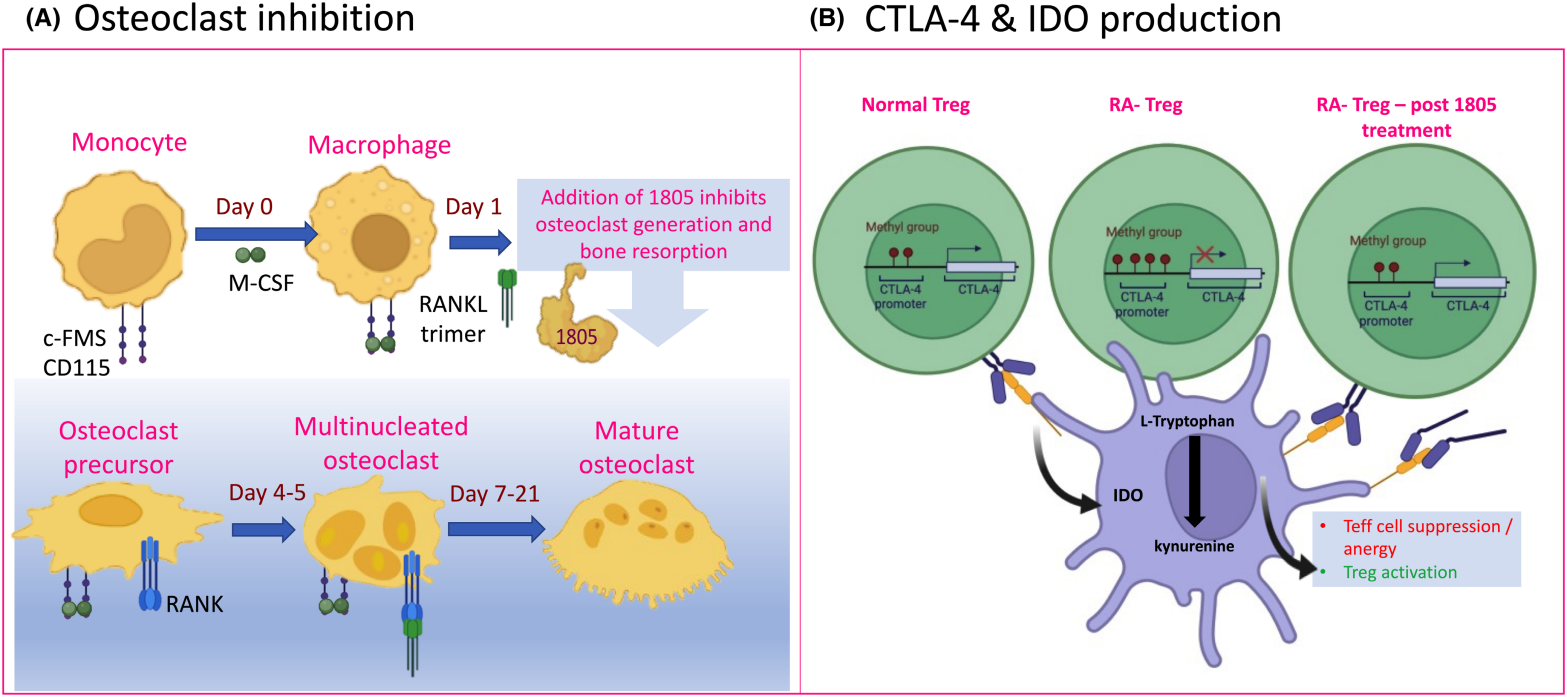
IRL201805 exposure to myeloid/lymphoid cells affects key immune‐regulatory pathways. (A) IRL201805 directly suppresses key cell surface receptors CD115/c‐FMS and RANK and nuclear translocation of NF‐κB by canonical and noncanonical pathways in response to both TNF‐α and RANKL, leading to suppression of human osteoclast formation^5^. (B) T‐regulator cells express CTLA‐4 on their cell surface; this is reduced in RA patients possibly by epigenetic factors^120^; however, treatment of Tregs with IRL201805 increases both cell surface and release of soluble CTLA‐4 into the circulation (Corrigall et al., paper in preparation).

Myeloid cells (monocytes and DCs) predominantly bind and internalize BiP or IRL201805. There are subsets of B cells and T cells that IRL201805 has been demonstrated to bind to directly.^96^ In the peripheral blood, IRL201805 is rapidly internalized by monocytes (Figure  2C), where IRL201805 has been shown to have a direct effect on various phenotypical and metabolic functions of myeloid cells. Osteoclast generation by cultured human monocytes is inhibited by BiP (Figure 3A) and bone resorption decreased as measured by lacunar formation on dentine slices.^5^ Corrigall et al. have shown that IRL201805 uptake leads to immunosuppressive characteristics in myeloid cells, such as lower surface expression of co‐stimulatory molecules CD83/86, lower HLA‐DR expression, increased IDO expression,^3^ increased secretion of IL‐10 and suppression of TNF‐α and IL‐1β release.^4^ The change in cytokines in part appears to be through regulated inhibition of NF‐κB, a regulator of cytokine production.^7^ These anti‐inflammatory features of extracellular IRL201805 help to regulated and resolve chronic inflammation.

We have consistently observed IRL201805 binding preferentially to classical, intermediate and nonclassical peripheral monocytes, but we have also observed that IRL201805 binds to distinct subsets of human‐derived DCs. This is important, as different DC subsets have distinctive functions in local immunosurveillance, migration and antigen presentation. This can influence their capacity to induce immune tolerance via their ability to express IDO and ultimately activate suppressive features of Tregs (see Figure 3B).

Corrigall et al. observed antibodies against GRP78/BiP in 30% of RA patient sera (*n* = 54) compared with 10% of control sera (*n* = 10).^38^ Detection of antibodies that react with self‐proteins indicates that despite normal mechanisms to reduce these cells, such as via physiological elimination (negative selection), functional inactivation (anergy) of high affinity and self‐reactive T/B lymphocytes in the thymus, some autoreactive cells with lower affinity are spared and persist in the peripheral immune system. Such antibodies are not necessarily pathogenic and may in fact be protective,^97^, ^98^, ^99^ circulating as low titre IgG autoantibodies in both control and disease subjects (Table 2). Some subsets of B cells appear to have regulatory properties such as CD38^+^ B cells. For example, CD38^+^ subset of B cells are capable of generating autoantibodies, but interestingly, CD19^high^IgDCD38^high^CD24^high^CD5^high^ are also known to produce IL‐10^100^ and are associated with the generation of Tregs.^101^ We have observed that IRL201805 can bind differentially to subsets of B cells that are currently being characterized.

Antigens, including self‐antigens (e.g. GRP78/BiP), can enter the APC receptor‐mediated endocytic pathway by accumulating in early endosomes.^102^ These vesicles then fuse with late endosomal–lysosomal antigen processing compartments. Within these compartments, proteins are proteolytically cleaved and some of the generated peptides complex with the HLA class II molecules and can migrate in these multi‐vesicles to the plasma membrane where they are recognized by CD4^+^ T cells.^103^ In autoimmune diseases, several HLA class II molecules are high‐risk biomarkers for autoimmunity. In RA, the dominant HLA class II locus that leads to disease susceptibility is the HLA‐DRB1 gene. This locus is highly polymorphic and has 2690 allele variants, encoding 1899 proteins^104^ and is encoded by six exons. Exon 2 is an extracellular domain of particular interest, as it is the hyper‐variable region 3 (HVR3) that contains the ‘shared epitope’ (SE) motifs thought to confer risk for developing RA. Approximately 62%–80% of RA patients have SE alleles compared with healthy controls (39%–52%).^105^ Shared epitope sequences allow the presentation of self‐antigens to T cells and thus play a key role in the development of RA.^106^ Therefore, SE‐containing alleles generating HLA proteins that are potential self‐binders to extracellular GRP78/BiP or IRL201805 are of interest.

Shoda and colleagues investigated the ability of full‐length GRP78/BiP or 20 mer peptides overlapping by five amino acids (10 μg/ml) to be taken up human APCs in PBMC cultures over 4 days and monitored peptide uptake T‐cell activation.^107^ In some experiments, they removed the CD25^+^ cells (to deplete Tregs) and found some of the suppressive properties diminished, for example IL‐10 production. They also examined the direct binding of GRP78/BiP peptides to solid‐phase bound extracellular 94 mer peptides of HLA–DRB1*0401, DRB1*0405 and HLA–DRA1*0101 molecules containing the SE sequences. They identified several GRP78/BiP peptides that caused varying degrees of T‐cell proliferation. The strongest binder of HLA‐DRB1 binding was GRP78/BiP^336–355^ with an IC_50_ of ~50 nM. GRP78/BiP^336–355^ (RSTMKPVQKVLEDSDLKKSD) induced the strongest proliferation in a SE‐dose‐dependent manner; SE+/+ > SE+/− > SE−/−. Ten additional peptides produced a very weak proliferative response and the GRP78/BiP^456–475^ peptide (DNQPTVTIKVYEGERPLTKD) was identified as an IL‐10‐inducing epitope in RA patients and healthy donors, that could be blocked by anti‐HLA blocking antibodies, suggesting T‐cell receptor interaction with the BiP peptide containing HLA molecule was essential for IL‐10 production. However, the IRL201805 protein is a potent anti‐inflammatory mediator.

In a separate study focused on Type 1 diabetes, citrullinated GRP78/BiP^R510^ containing peptides have been shown by Buitinga and co‐workers^108^ to bind to the shared epitope HLA‐DRB1*04:01 molecules in postcytokine‐treated human islets from type 1 diabetes patients, which were associated with the identification of higher CD4^+^ T‐cell frequencies directed against citrullinated GRP78 (citGRP78) epitopes. In this study, several native and citrullinated GRP78/BiP peptides were identified as binding to the DRB1*0401. In addition, autoantibodies against citCRP78/BiP were identified in a subset of patients, providing further evidence that APC‐processed GRP78/BiP peptides evoked CD4+ T‐cell and B‐cell responses.

## 4 THERAPEUTIC APPLICATIONS

### 4.1 Importance of chaperone location and concentration

**FIGURE 4:**
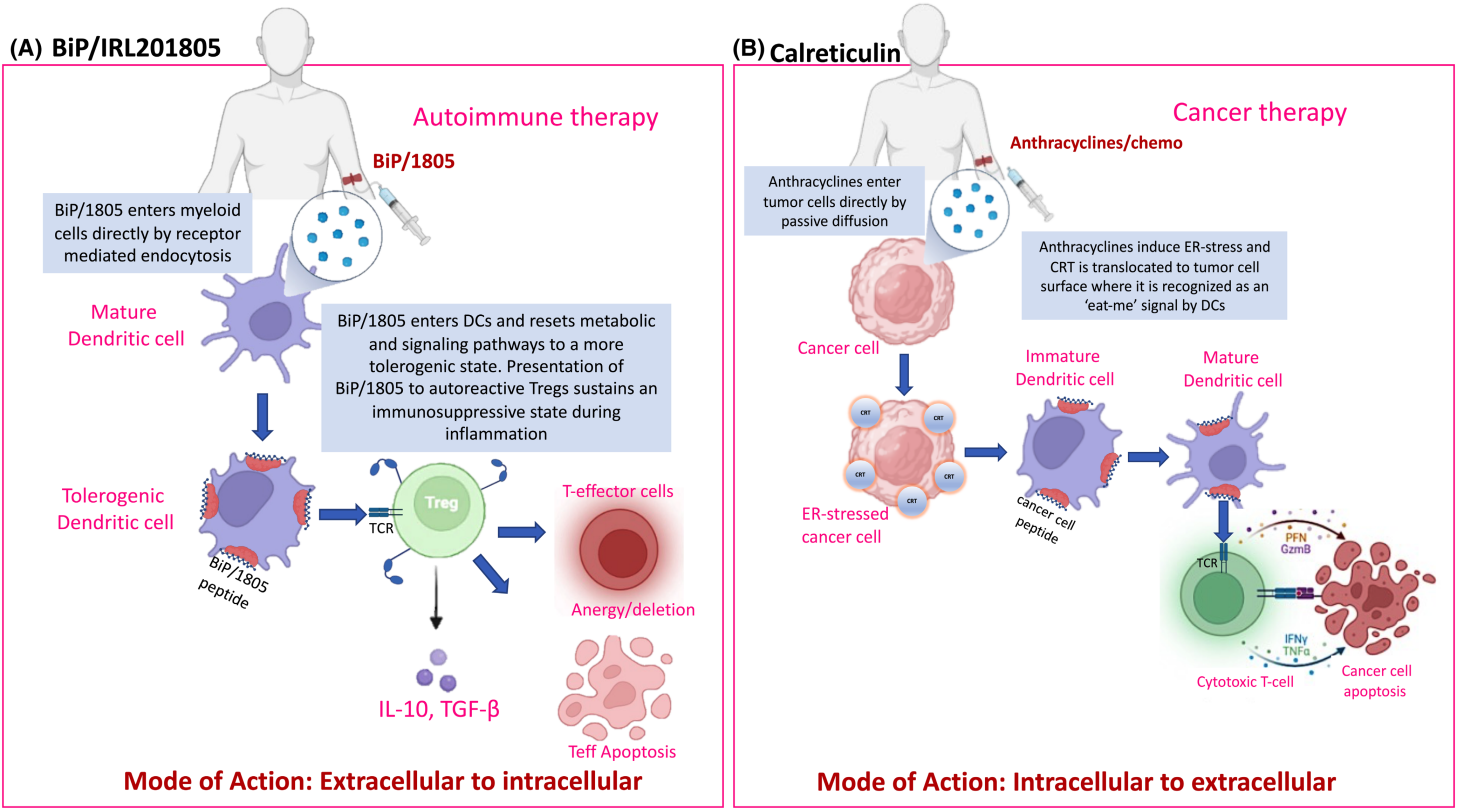
In human therapeutic trials, the way IRL201805 or calreticulin is presented to dendritic cells governs their downstream immune cell signalling. (A) With infusion of IRL201805, it is rapidly taken up mainly by myeloid cells, including dendritic cells (DCs), leading to sustained tolerogenic changes in the DCs and possibly presentation of IRL201805 peptides to ‘primed’ Tregs which can direct immunosuppressive responses against autoimmune effector cells. (B) Calreticulin is not infused directly, but endoplasmic reticulum (ER) stress‐inducing drugs such as anthracyclines in combination with chemotherapeutics target tumour cells leading to anterograde translocation of wildtype CRT to the surface of ER stress cancer cells, which acts as an ‘eat‐me signal to immature DCs, which rapidly mature and signal to cytotoxic T cells to destroy the cancer cells.

For both IRL201805 and CRT to work therapeutically, it appears their interaction with immune cells is quite different. For IRL201805, extracellular delivery of the protein to immune cells can lead to tolerogenic changes in myeloid cells (monocytes/macrophages, DCs), which can also promote regulatory T‐cell responses in inflammatory environments. By contrast, endogenous translocation of CRT to the cell surface of tumour cells promotes a DC inflammatory response that can promote a cytotoxic T‐cell response against tumours (Figure 4B). For both these chaperones, the natural levels of plasma endogenous GRP78/BiP or the surface expression of CRT on cells is most likely suboptimal to provoke an optimal immune response. Therapeutic interventions have shown that increased concentration of these proteins can have clinical benefit.

### 4.2 Therapeutic evidence for extracellular for CRT

The therapeutic potential of CRT has been examined in a number of ways: (a) cancer chemotherapeutics and PDT can induce ER stress leading the anterograde translocation of CRT to the cell surface of some tumours where it can affect both innate and adaptive immunity, (b) recombinant CRT has been shown to have a beneficial role in wound healing^109^ and (c) the chaperone properties of CRT have been exploited to enhance antigen therapeutic vaccine development.^110^

The biggest challenge is being able to manipulate CRT expression on the surface of pre‐apoptotic cells. Can for instance ICD be regulated in a controlled manner to act as an effective therapeutic intervention? The concern arising from inducing ‘secreted’ CRT is that it can act as a decoy and bind to DCs directly, preventing or competing with the CRT‐coated tumour cells from being recognized and phagocytosed.^69^ There is conflicting evidence as to the benefit of soluble CRT as an adjuvant to aid cancer treatments. In a photodynamic therapy (PDT)‐treated squamous cell carcinoma murine immune‐competent model, recombinant calreticulin (400 μg/mouse) injected peritumourally immediately after PDT was found to bind to mouse SCCVII tumour cells treated by PDT and rendered a significant improvement in tumour response to PDT from marginally curative to the solid levels of about 40% cure rates.^111^ In a C57BL/6 mouse model, intravenous injection of 200 μg rCRT +/− MCA‐205 fibrosarcoma‐derived cells treated with 300 μM oxaliplatin, or MTX (4 μM) inhibited selective phagocytosis of CRT‐exposing tumour cells both in vitro and in vivo, but not bacteria, demonstrating the phagocytic pathway in general is not inhibited by soluble CRT, but the uptake of tumour cells by DCs is inhibited.^69^ Evidently different cancer treatments influence the recognition and removal of tumour cells by DCs in the presence of extracellular CRT.

As a cancer treatment, pre‐apoptotic surface expression of CRT is not a treatment but might promote immunogenic recognition and targeting of some tumour cells. Chemotherapeutic agents with different modes of action, for example anthracyclines (insertion of drug into nucleic acid) and taxanes (microtubule disruption) produce ER stress as a downstream effect and promote cell surface display of CRT. As part of the toxic stress pathways induced by chemotherapeutics, other ICD inducers are also released such as adenosine triphosphate, annexin A1, high‐mobility group B1 and type‐1 interferons. Which of these plays the most significant role in ICD is difficult to measure in patients who respond to such treatments. The quantification of how much tumour cell surface CRT is required, or requirement of other ICD inducers to aid tumour eradication is difficult to gauge. Too much or too little of an ICD inducer may influence the efficacy of tumour therapeutics. A classic example of this is the concentration of extracellular ATP during cancer therapy.^112^

ATP can be an ICD inducer that can be released from cells actively by cell stress or pharmacological treatment. Extracellular ATP is hydrolysed to adenosine by ectoenzymes CD73 and CD39,^113^ where its biological activity can promote Treg‐mediated immunosuppression. While an extracellular concentration of ATP ≤250 nM does not modulate Treg function, a 4000‐fold increase in extracellular ATP concentration (1 mM) triggers Treg immunoregulatory capabilities.^114^ This suggests involvement of a relatively insensitive receptor with low affinity for ATP metabolites thus requiring higher concentrations of ATP before it can signal via its receptor. The importance of an ATP‐ATP receptor interaction is illustrated in a murine acute myeloid leukaemia (AML) model. When AML cells are treated with the chemotherapeutic anthracycline–daunorubicin, they released ATP, upon co‐cultured with DCs; the DCs upregulate IDO production that in turn induced antileukaemic Tregs. However, the action of daunorubicin failed to induce antitumour Tregs in mice lacking the ATP receptor P2RX7.^112^ The presence of ATP in extracellular space acts as ‘find me’ signal, being a chemoattractant for DCs precursors. ATP binds to the P2RX7 receptor on DCs triggering IDO‐mediated tolerance to Tumour cells. In this regard, ATP and its metabolites are immune checkpoint regulators in cancer. Several therapeutic strategies are being proposed to impede extracellular adenosine metabolism.^115^

Similarly, if soluble CRT is released from tumour cells, it can inhibit phagocytosis of CRT‐coated cells both in vitro and in vivo. Increased exposure of CRT on malignant cells is associated with therapy‐relevant adaptive immune responses and superior therapeutic outcome in solid tumours and haemato‐oncological diseases because surface‐exposed CRT acts as an ‘eat‐me’ signal facilitating the phagocytosis of stressed and dying cancer cells by immature DCs, thus favouring antitumour immune responses. Soluble mutant CRT has been detected in the plasma^51^, ^58^, ^69^ (Table 1); Liu and co‐workers have shown that soluble CRT inhibits the phagocytosis of cancer cells by DCs, thus dampening anticancer immune responses. Furthermore, systemic elevations of soluble CRT secreted from tumours or that is artificially supplied by injection of the recombinant protein decreased the efficacy of immunotherapy. Thus, depending on its location, CRT can have immunostimulatory or immunosuppressive functions.

### 4.3 Therapeutic evidence for extracellular BiP/1805

Work from a number independent laboratories provides evidence that GRP78/BiP is a self‐antigen that is rapidly taken up by APCs, binds to specific HLA class II molecules and can trigger GRP78/BiP‐reactive Treg responses,^7^, ^107^, ^108^ that suppress Teff inflammatory responses against self‐antigens. We have identified several potent anti‐inflammatory changes in immune cells in response to IRL201805 that may allow a better understanding of regulation of inflammation pathway in RA patients and models of autoimmunity.^3^, ^4^, ^5^, ^6^, ^7^, ^38^, ^96^, ^116^, ^117^ Upon infusion of IRL201804 in RA patients, those individuals responsive to IRL201805 in terms of sustained reduction in DAS28‐ESR score and significant reductions in IL‐8 and VEGF reveal changes in myeloid cell functions. Preclinical work using a xenogeneic model, where human RA synovial tissue was transplanted into severe combined immunodeficient mice, demonstrated that mice treated with BiP/1805 showed reduced tissue expression of HLA‐DR, CD86 and production of IL‐6 and TNF‐α.^118^ Despite an increase in IL‐10 not been detected, the application of a neutralizing anti‐IL‐10 antibody negated the anti‐inflammatory properties of BiP/1805.

Previously using the CIA murine model, we had shown that adoptive transfer of BiP/1805‐treated splenocytes and lymph node cells, in the absence of additional protein, transferred the prophylactic and/or therapeutic properties of BiP/1805.^119^ This suggested a novel dissociation between the pharmacokinetics and pharmacodynamics, which was replicated in the RAGULA clinical trial, where a single infusion of IRL201805, led to a sustained reduction in inflammatory mediators and prolonged low disease activity in some patients.^117^

In the preclinical studies, there was evidence of direct immunomodulatory effect on myeloid cells with the upregulation of IDO and inhibition of NFκB activation and downstream inflammatory cytokine production such as TNF‐⍺, which in turn supported a Treg response, including increases in IL‐10 and CTLA‐4 and release of soluble CTLA‐4 (Corrigall et al., paper in preparation; Figure 3B). This is of interest because it has been previously shown that RA Tregs have an inability to induce the activation of the tryptophan‐degrading enzyme IDO and CTLA‐4 production is reduced, possibly by increased methylation of the CTLA‐4 promoter in RA Tregs.^120^ These multiple changes in regulatory molecules in both myeloid and lymphocytic cells provide compelling evidence that IRL201805 acts to fundamentally reset immune responses. In the RAGULA clinical trial, the responsiveness to IRL201805 treatment was most striking in patients who had higher levels of basal natural GRP78/BiP (Data not shown) in their circulation prior to IRL201805 infusion. Within 2 weeks of infusion, patients in this responsive group had significantly lower serum levels of C‐reactive protein, vascular endothelial growth factor and interleukin (IL)‐8 serum levels than those of the placebo group.^117^ We hypothesize that administrating pharmacological levels of IRL201805 can promote a greater immunosuppressive activity in APCs (monocyte/macrophage, DCs), which, in turn, activate Tregs that are primed to respond to self‐antigen (GRP78/BiP) aiding resolution of inflammation during autoimmunity (Corrrigall et al., paper in preparation). Once the inflammatory processes are resolved, the immune system would be expected to return to immune homeostasis via changes in stress, redox and metabolic signals, maintaining a balance between tolerance and immunogenicity. This approach differs from current therapeutics that tend to be immunosuppressive in nature (e.g. NSAIDs, steroids, biologics and JAK inhibitors).

### 4.4 Release of endogenous GRP78/BiP and CRT: Evidence of autoantibody generation

With the development of any therapeutic, natural or synthetic, the presence of antidrug antibodies and possible complications that may arise from their generation have to be assessed.

In relation to GRP78/BiP and CRT as naturally occurring intracellular proteins, they are somewhat protected from immune surveillance under normal physiological conditions. However, under certain pathological/stress conditions, autoantibodies against endogenous GRP78/BiP and CRT have been detected in several disease states compared with control subjects (see Table 2). The generation of anti‐GRP78/BiP or anti‐CRT antibodies may act as a biomarker of protein release from stress cells, but it should be remembered that the presence of autoantibodies is generally quite benign. Very few are disease‐specific or even pathologic and the majority play more of a part in diagnosis than therapy. As people age, the presence of autoantibodies in serum increases in variety and quantity without much harm to the healthy person. This occurs because proteins are continually being encountered by the immune system. However, if they cross‐react with part of an endogenous protein, it is possible that they will generate autoantibodies. Such antibodies would only be of concern if they neutralized the effects of cell surface CRT in tumour resolution or altered extracellular IRL201805 efficacy in resetting autoimmune diseases. This conjecture requires further examination. In some cases, presentation of antigens to generate autoreactive immunosuppressive Tregs and generation of B cells that recognize self‐antigens can be beneficial in preventing autoimmunity.^121^ There is a fine balance between activating some lymphocyte subsets and inhibiting others. What is clear is that both membrane‐bound CRT and the extracellular manufactured modified homologue of GRP78/BiP ‐ IRL201805 can provoke physiologically relevant immune‐modulating effects in selected diseases.

## 5 CONCLUDING REMARKS

It has become clear over a number of decades that the ~20,000 protein‐encoding genes in the human body, depending on how they are transcribed and the splice variants produced and post‐translationally modifications that arise can ultimately generate ~70,000 proteins.^122^ Not surprisingly, many proteins have been revealed to have more than one function.^123^ Then, each cell could contain up to 42 million individual protein molecules.^124^ Chaperones more than most proteins have to be adaptable; they must be able to function in different redox conditions and have effective ion buffering capacity, bind to and disengage from peptides, folded and unfolded protein, help glycosylate secretory proteins with other specialist quality control proteins and then shuttle between organelles and occasionally to the cell surface and beyond.^125^, ^126^ In each location, they appear to have a different function. Once outside cells, chaperones switch from protein folding proteins to DAMPs (e.g. CRT) or regulatory‐associated molecular patterns—RAMPs (e.g. GRP78/BiP).

As new generations of high‐tech ‘omics‐driven’ therapeutics are developing, Biotech and Pharma industries strive to generate engineered cells as bespoke therapies for a multitude of diseases. By contrast, these resolution‐promoting chaperones, for example CRT and IRL201805, may herald a new generation of biologics affecting multiple pathways common to inflammatory diseases and thereby unlock some of the multifaceted immune‐regulatory qualities of these highly conserved proteins.

## AUTHOR CONTRIBUTIONS

**Paul Eggleton:** Conceptualization (equal); data curation (equal); formal analysis (equal); funding acquisition (equal); investigation (equal); methodology (equal); project administration (equal); software (equal); supervision (equal); validation (equal); visualization (equal); writing – original draft (equal); writing – review and editing (equal). **Jorge De Alba:** Validation (equal); writing – original draft (equal); writing – review and editing (equal). **Michael Weinreich:** Writing – original draft (equal); writing – review and editing (equal). **Perry Calais:** Formal analysis (equal); resources (equal); validation (equal); writing – original draft (equal); writing – review and editing (equal). **Roly Foulkes:** Conceptualization (equal); funding acquisition (equal); project administration (equal); writing – original draft (equal); writing – review and editing (equal). **Valerie M. Corrigall:** Conceptualization (equal); data curation (equal); formal analysis (equal); funding acquisition (equal); investigation (equal); methodology (equal); supervision (equal); writing – original draft (equal); writing – review and editing (equal).

## CONFLICT OF INTEREST

The authors confirm that there are no conflicts of interest regarding the calreticulin research cited in this article. All authors are employees or consultants of Revolo Biotherapeutics Limited, https://revolobio.com.

## Supporting information


Appendix S1.

## Data Availability

The data cited in this article are openly available in a public repository that issues datasets with DOIs and all information with DOIs are listed in the reference section of the article.
