# Supplementary material for: The therapeutic mavericks: Potent immunomodulating chaperones capable of treating human diseases
Source: J Cell Mol Med. 2023 Jan 18;27(3):322–39. doi: 10.1111/jcmm.17669 (PMC9889696; doi:10.1111/jcmm.17669)
Supplement: Supplementary file 1 — Appendix S1. [file JCMM-27-322-s001.docx]

**Supplementary Table 1**. Definitions of abbreviations used in this article.

| **Abbreviation** | **Definition** |
| --- | --- |
| **ATF6** | **Activating transcription factor 6α/β,** activator of the unfolded protein response pathway |
| **BAK1** | **BCL2 Antagonist/Killer 1**, apoptotic regulator |
| **BiP/1805** | Extracellular human recombinant manufactured modified BiP (IRL201805) that has been used in phase I/IIA RA clinical trial |
| **BAX** | **BCL2 Associated X**, apoptotic regulator |
| **COPI** | **Coat protein complex I,** transports proteins back to ER |
| **COPII** | **Coat protein complex II,** transports proteins fro ER to Golgi, occasionally to plasma membrane via anterograde transport |
| **DAMPs** | **Damage‐associated molecular patterns,** endogenous danger molecules released from cells that activate immune responses (e.g. CRT) |
| **DAS28-ESR** | **Disease activity score 28** erythrocyte sedimentation rate, clinical score of joint tenderness/swelling |
| **GRP78/BiP** | **Glucose-regulated protein 78/binding-immunoglobulin protein**, intracellular ER-chaperone, detected on cell surface and plasma during at low levels during cell stress |
| **HLA-DR** | **Human leukocyte antigen DR (HLA)** is a MHC II receptor on antigen presenting cells that presents foreign and self-antigens to T-cells via their T cell receptor |
| **ICD** | **Immunogenic cell death** is a regulated form of immune-mediated killing of tumor cells by recognizing damage-associated molecular patterns on tumor cell surfaces |
| **IRL201805** | **A good manufacturing practice form of human GRP78/BiP** used in clinical trials. Nb. In figures, IRL201805 is abbreviated to 1805 for brevity. |
| **IRE1** | **Inositol-requiring kinase/endonuclease,** activator of the unfolded protein response pathway |
| **KDEL** | **Lysine-Aspartic acid-Glutamic acid-Leucine peptide sequence** located on the C-terminal end of proteins (e.g., PDI, BiP & CRT) |
| **PERK** | **Protein kinase-like ER kinase**, activator of the unfolded protein response pathway |
| **RAMPs** | **Regulatory-associated molecular patterns** – (e.g., BiP/1805) taking up by APCs inducing tolerogenic immune responses |
| **SNARE** | **Soluble N-ethylmaleimide-sensitive-factor attachment protein receptor**, involved in membrane fusion |
| **Teff** | **Effector T-cell** – pro-inflammatory  CD4^+ve^CD25^-ve^CD127^hi^FoxP3^-ve^ |
| **Treg** | **Regulatory T-cell** – anti-inflammatory  CD4^+ve^CD25^+ve^ CD127^lo^FoxP3^+ve^ |
